# Supplementary material for: Neuronal connected burst cascades bridge macroscale adaptive signatures across arousal states
Source: Nat Commun. 2023 Oct 27;14:6846. doi: 10.1038/s41467-023-42465-2 (PMC10611774; doi:10.1038/s41467-023-42465-2)
Supplement: Supplementary file 1 — Supplementary Information [file 41467_2023_42465_MOESM1_ESM.pdf]

Supplementary Figures

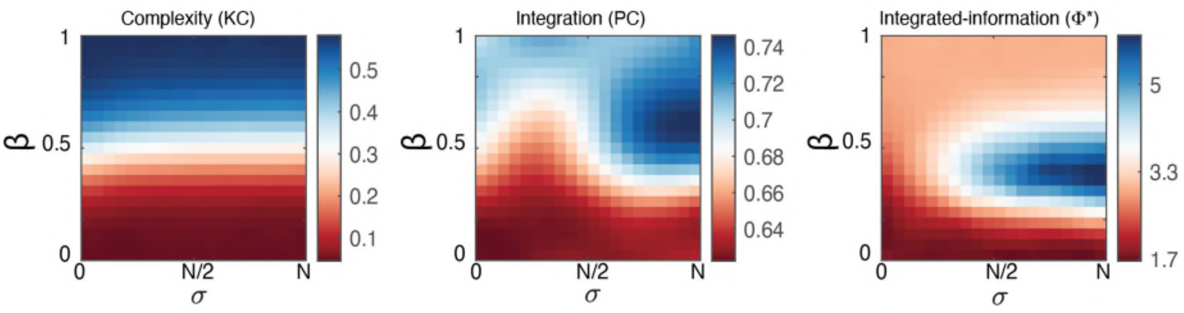

Fig. S1: Comparison of the three complex, adaptive signatures calculated using a spatially overlapping Gaussian smoothed coarse-sampling.

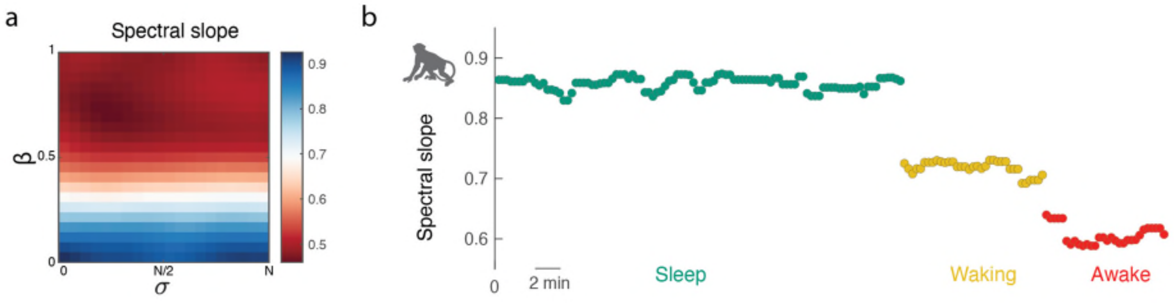

Fig. S2: (a) Spectral slope across the model state space. **(b)** spectral slope calculated across a macaque recording (Fig. 3a).

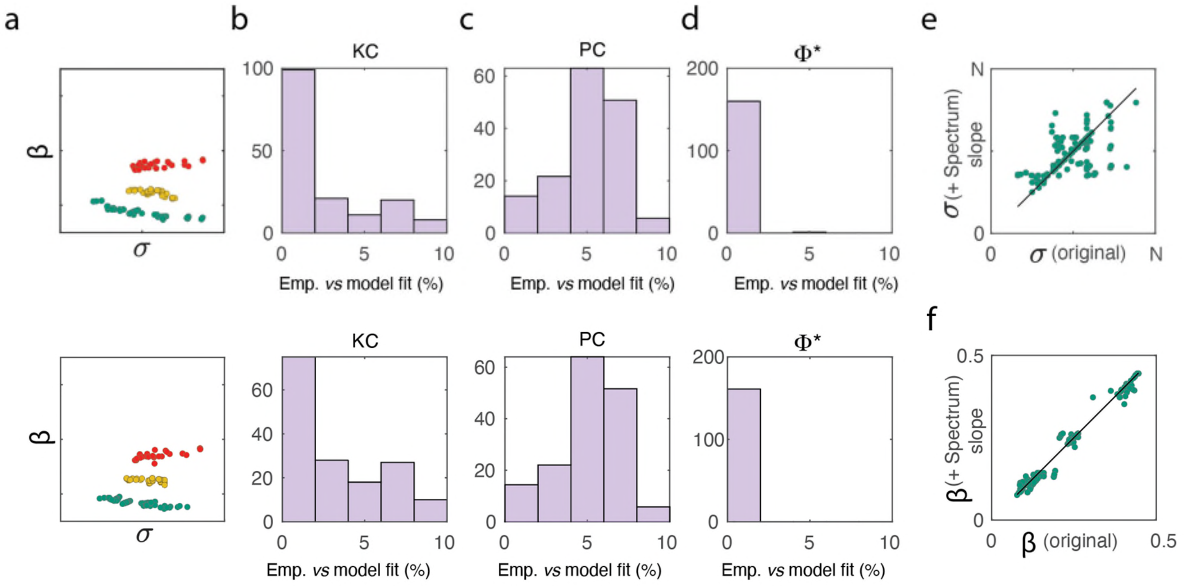

Fig. S3: (a) Comparison of the swarm fitting using (KC,PC, Φ^*; top) and (KC,PC, Φ^*, and spectra slope; bottom). **(b-d)** the optimisation fits each signature within 10% of the empirically measured value. **(e-f)** Including an extra parameter (spectrum slope) subtly shifts σ **(e)** and does not change β significantly **(f)**.

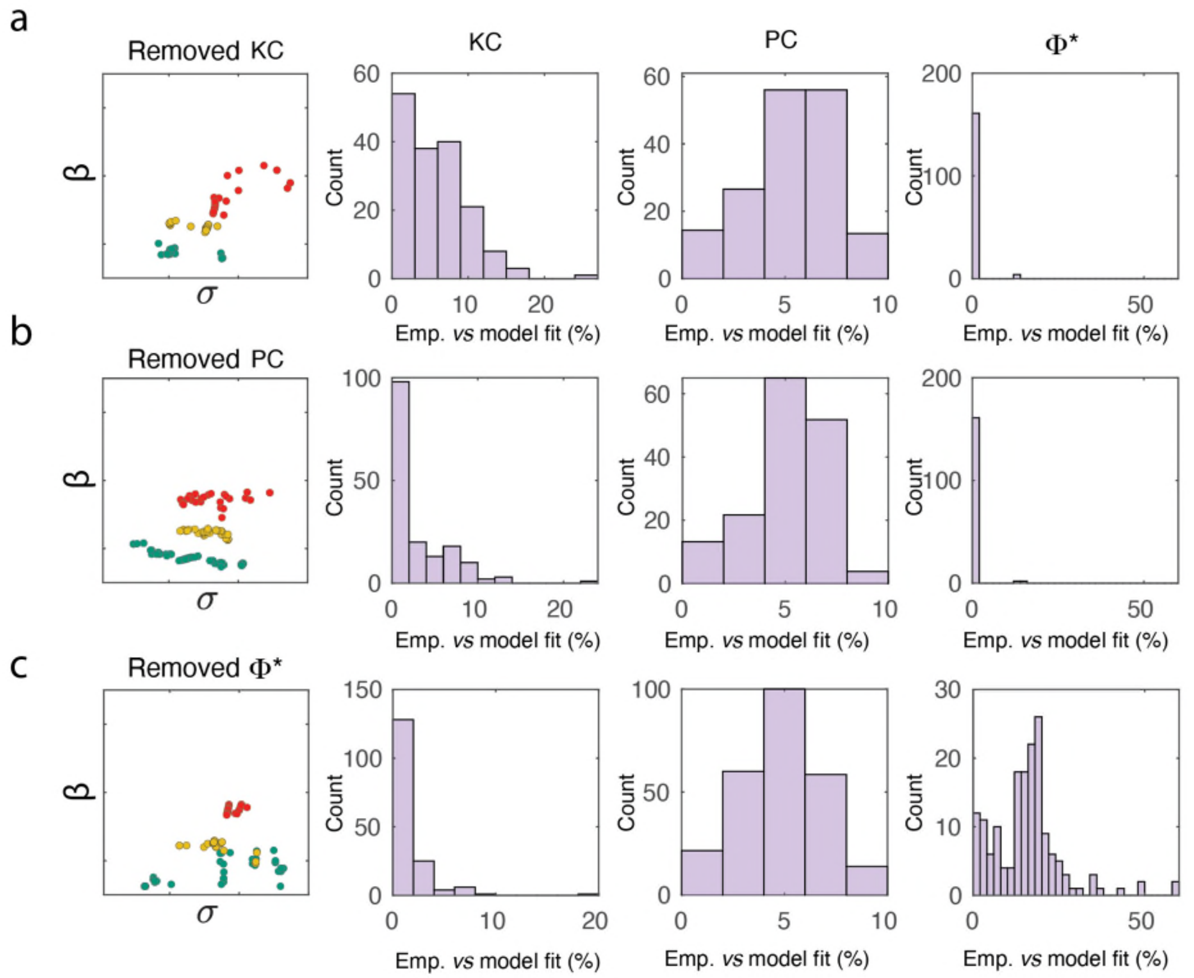

Fig. S4: Comparison of model fits on a macaque recording (Fig. 3a) only using two complex, adaptive signatures. **(a)** Model state-space (left) and match with empirical measurements (right) after removing KC. **(b)** Model state-space (left) and match with empirical measurements (right) after removing PC. **(c)** Model state-space (left) and match with empirical measurements (right) after removing Φ^*.

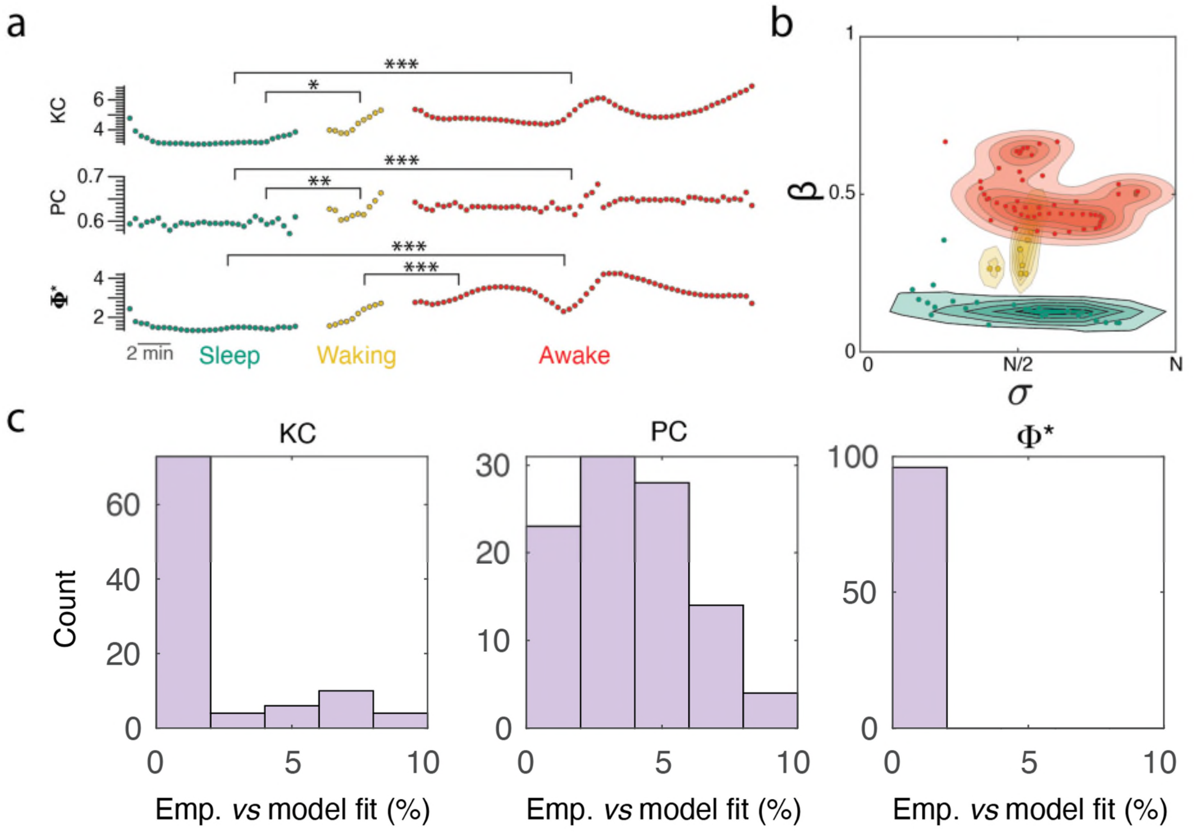

Fig. S5: (a) In a separate macaque EGoG recordings from Fig. 2a, the three complex, adaptive signatures significantly change across 20s epochs of arousal from sleeping (teal) to awake (red). **(b)** Inverted location of each 20s epoch in the model state space following hybrid particle swarm/convex optimisation minimising the difference between model and empirical complex, adaptive dynamics where the clouds are five evenly spaced contour lines (2% to 98%) of the probability density estimate for each state. **(c)** Fit quality (percentage error difference) between model estimated and empirically measured complex, adaptive signatures. Statistical significance across empirical complex, adaptive dynamics denoted by * $p < 0.05$, ** $p < 0.01$, and *** $p < 0.001$ Kruskal-Wallis multiple comparison tests; see Table 1 for p-values.

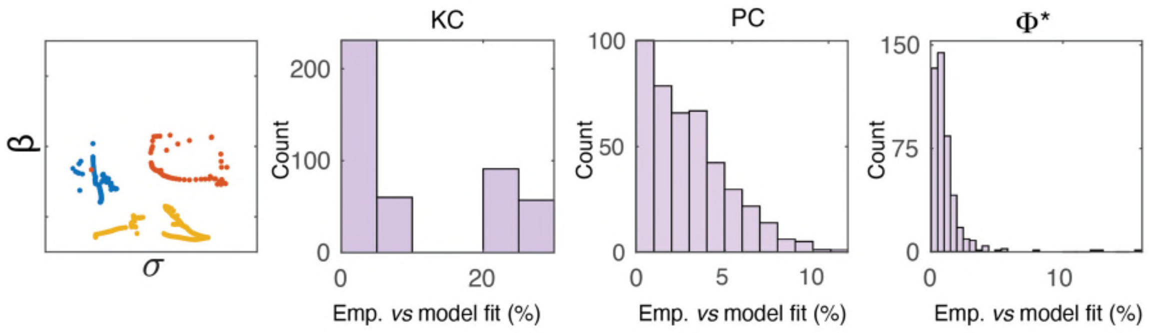

Fig. S6: Match (percentage difference) between model estimated and empirically measured complex, adaptive signatures in the human EEG recordings.

Figure	Comparison	P value (Kruskal-Wallis multiple comparison corrected)
Fig. 3a - KC	Sleep vs Waking	4.5×10^{-12}
Fig. 3a - KC	Sleep vs Awake	1.8×10^{-16}
Fig. 3a - KC	Waking vs Awake	0.01
Fig. 3a - PC	Sleep vs Waking	2.3×10^{-8}
Fig. 3a - PC	Sleep vs Awake	1.9×10^{-21}
Fig. 3a - PC	Waking vs Awake	0.002
Fig. 3a - Φ^*	Sleep vs Waking	1.7×10^{-12}
Fig. 3a - Φ^*	Sleep vs Awake	1.8×10^{-24}
Fig. 3a - Φ^*	Waking vs Awake	0.02
Fig. 3d - KC	Sleep vs Waking	0.15
Fig. 3d - KC	Sleep vs Awake	1×10^{-32}
Fig. 3d - KC	Waking vs Awake	1×10^{-32}
Fig. 3d - PC	Sleep vs Waking	1×10^{-32}
Fig. 3d - PC	Sleep vs Awake	5×10^{-20}
Fig. 3d - PC	Waking vs Awake	1×10^{-32}
Fig. 3d - Φ^*	Sleep vs Waking	2.6×10^{-21}
Fig. 3d - Φ^*	Sleep vs Awake	1×10^{-32}
Fig. 3d - Φ^*	Waking vs Awake	5×10^{-8}
Fig. S5 - KC	Sleep vs Waking	0.02
Fig. S5 - KC	Sleep vs Awake	4×10^{-15}
Fig. S5 - KC	Waking vs Awake	0.06
Fig. S5 - PC	Sleep vs Waking	0.01
Fig. S5 - PC	Sleep vs Awake	1×10^{-15}
Fig. S5 - PC	Waking vs Awake	0.07
Fig. S5 - Φ^*	Sleep vs Waking	0.15
Fig. S5 - Φ^*	Sleep vs Awake	1×10^{-16}
Fig. S5 - Φ^*	Waking vs Awake	0.001

Supplementary Table 1: Exact p values for significance testing of complex, adaptive dynamics across arousal states.
