# Supplementary material for: Neuronal connected burst cascades bridge macroscale adaptive signatures across arousal states
Source: Nat Commun. 2023 Oct 27;14:6846. doi: 10.1038/s41467-023-42465-2 (PMC10611774; doi:10.1038/s41467-023-42465-2)
Supplement: Supplementary file 3 — Reporting Summary [file 41467_2023_42465_MOESM3_ESM.pdf]

nature portfolio

Corresponding author(s): Munn

Last updated by author(s): 10.10.23

Reporting Summary

Nature Portfolio wishes to improve the reproducibility of the work that we publish. This form provides structure for consistency and transparency in reporting. For further information on Nature Portfolio policies, see our Editorial Policies and the Editorial Policy Checklist.

Statistics

For all statistical analyses, confirm that the following items are present in the figure legend, table legend, main text, or Methods section.

n/a	Confirmed	
[ ]	[x]	The exact sample size (*n*) for each experimental group/condition, given as a discrete number and unit of measurement
[ ]	[x]	A statement on whether measurements were taken from distinct samples or whether the same sample was measured repeatedly
[ ]	[x]	The statistical test(s) used AND whether they are one- or two-sided<br>*Only common tests should be described solely by name; describe more complex techniques in the Methods section.*
[x]	[ ]	A description of all covariates tested
[ ]	[x]	A description of any assumptions or corrections, such as tests of normality and adjustment for multiple comparisons
[ ]	[x]	A full description of the statistical parameters including central tendency (e.g. means) or other basic estimates (e.g. regression coefficient) AND variation (e.g. standard deviation) or associated estimates of uncertainty (e.g. confidence intervals)
[ ]	[x]	For null hypothesis testing, the test statistic (e.g. *F*, *t*, *r*) with confidence intervals, effect sizes, degrees of freedom and *P* value noted<br>*Give P values as exact values whenever suitable.*
[x]	[ ]	For Bayesian analysis, information on the choice of priors and Markov chain Monte Carlo settings
[x]	[ ]	For hierarchical and complex designs, identification of the appropriate level for tests and full reporting of outcomes
[ ]	[x]	Estimates of effect sizes (e.g. Cohen's *d*, Pearson's *r*), indicating how they were calculated

Our web collection on statistics for biologists contains articles on many of the points above.

Software and code

Policy information about availability of computer code

Data collection	All model simulations were conducted in MATLAB (2020a) with custom code available at https://github.com/Bmunn/Layer5_Arousal. ECoG and EEG data collection are described in their accompanying manuscripts. High-density EEG data were collected using a NA300 EGI system with 256-channel gel caps. Electrodes were manually prepared with application of electrolyte gel to achieve electrode impedances <50 kΩ. Data were recorded using EGI Net Station Acquisition 5.4 software (Eugene, OR, USA). ECoG signals were recorded at a sampling rate of 1 kHz by a Cerebus data acquisition system (Blackrock, UT, USA).
Data analysis	All analysis was conducted in MATLAB (2020a) with custom code available at https://github.com/Bmunn/Layer5_Arousal

For manuscripts utilizing custom algorithms or software that are central to the research but not yet described in published literature, software must be made available to editors and reviewers. We strongly encourage code deposition in a community repository (e.g. GitHub). See the Nature Portfolio guidelines for submitting code & software for further information.

Data

Policy information about availability of data

All manuscripts must include a data availability statement. This statement should provide the following information, where applicable:

- Accession codes, unique identifiers, or web links for publicly available datasets
- A description of any restrictions on data availability
- For clinical datasets or third party data, please ensure that the statement adheres to our policy

The data generated in this study are provided in the Source Data file. The macaque ECoG data can be obtained from (www.www.neurotycho.org/sleep-task, 2 male

macaques Chibi/George). The human EEG data is available under restricted access, access can be obtained following approval by the institutional review board at the University of Wisconsin-Madison.

Human research participants

Policy information about studies involving human research participants and Sex and Gender in Research.

Reporting on sex and gender	n = 20, 5 female
Population characteristics	Participants were healthy volunteers between 18 and 40 yr old without prior contraindications to anaesthetics
Recruitment	Subjects were enrolled in the Understanding Consciousness Connectedness and Intraoperative Unresponsiveness Study (NCT03284307).
Ethics oversight	University of Wisconsin, Madison

Note that full information on the approval of the study protocol must also be provided in the manuscript.

Field-specific reporting

Please select the one below that is the best fit for your research. If you are not sure, read the appropriate sections before making your selection.

[x] Life sciences [ ] Behavioural & social sciences [ ] Ecological, evolutionary & environmental sciences

For a reference copy of the document with all sections, see nature.com/documents/nr-reporting-summary-flat.pdf

Life sciences study design

All studies must disclose on these points even when the disclosure is negative.

Sample size	No statistical method was used to predetermine sample size
Data exclusions	No data were excluded from the analyses
Replication	Replication was repeated between different animals and repeated measurements taken across and between individuals
Randomization	The experiments were not randomised
Blinding	Data was not analysed or collected with respect to groupings. All recordings across individuals are analysed.

Reporting for specific materials, systems and methods

We require information from authors about some types of materials, experimental systems and methods used in many studies. Here, indicate whether each material, system or method listed is relevant to your study. If you are not sure if a list item applies to your research, read the appropriate section before selecting a response.

Materials & experimental systems

n/a	Involved in the study	
[x]	[ ]	Antibodies
[x]	[ ]	Eukaryotic cell lines
[x]	[ ]	Palaeontology and archaeology
[x]	[ ]	Animals and other organisms
[ ]	[x]	Clinical data
[x]	[ ]	Dual use research of concern

Methods

n/a	Involved in the study	
[x]	[ ]	ChIP-seq
[x]	[ ]	Flow cytometry
[x]	[ ]	MRI-based neuroimaging

Clinical data

Policy information about clinical studies

All manuscripts should comply with the ICMJE guidelines for publication of clinical research and a completed CONSORT checklist must be included with all submissions.

Clinical trial registration	NCT03284307
Study protocol	https://clinicaltrials.gov/ct2/show/NCT03284307
Data collection	Subjects (n = 20, 5 female) were enrolled in the UNderstanding Consciousness Connectedness and Intra-Operative Unresponsiveness Study (UN-ConsCIOUS, NCT03284307). Participants were healthy volunteers between 18 and 40 years old without prior contraindications to anaesthetics. Sex and/or gender was not considered in the study design. All subjects provided written consent for each study visit and data were collected in accordance with a protocol approved by the institutional review board at the University of Wisconsin-Madison.<br>Actual Study Start Date : August 10, 2017<br>Actual Primary Completion Date : March 12, 2020<br>Actual Study Completion Date : March 12, 2020
Outcomes	Incidence of disconnected conscious experience (dreaming) vs connected conscious experience (awareness of external world). [Time Frame: Intraoperative (During sedation-- up to 8 hours)]<br>The incidence of disconnected conscious experience (dreaming) versus connected conscious experience (awareness of the external world) during sedation is measured by subject self-report at the time of researcher initiated inquiry.
